# P-1946. Epidemiology and Factors Associated with SARS-CoV-2 Transmission Among Students Following Resumption of Face-to-Face Classes in a Private University in Metro Manila, Philippines

**DOI:** 10.1093/ofid/ofae631.2105

**Published:** 2025-01-29

**Authors:** Dianne Hazel P Do, Jay Ron O Padua

**Affiliations:** University of Santo Tomas Hospital, Manila, National Capital Region, Philippines; University of Santo Tomas Hospital, Manila, National Capital Region, Philippines

## Abstract

**Background:**

Coronavirus disease (COVID-19) has brought disruptions in our lives including the education system. In the Philippines, the education system has transitioned to online and modular distance learning delivery of instruction due to the pandemic. To date, there has been no local study on the clustering rates within schools in the Philippines following resumption of face-to-face classes. This study aims to describe the demographic and clinical characteristics of students with confirmed COVID-19 and to determine the incidence of clustering among students after resumption of face-to-face classes.

**Figure:**
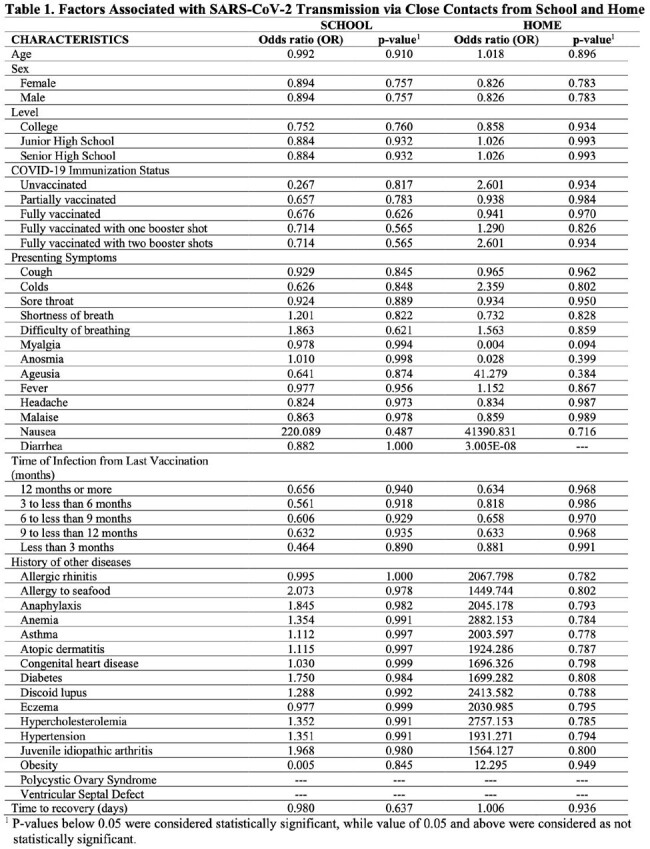

**Methods:**

A retrospective cross-sectional study on high school and college students of a private university with confirmed COVID-19 during the Academic Years (A.Y.) 2021-2022 and 2022-2023. Data was collected through digital medical charts review and analysis done using real statistics, multiple logistic regression model and cluster analysis.

**Figure:**
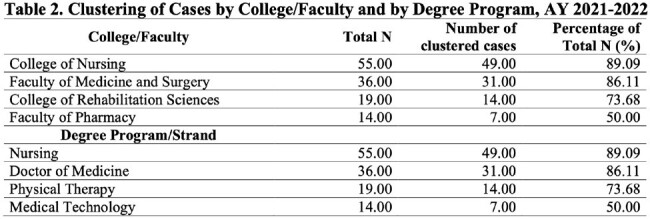

**Results:**

More female students were affected than male students in the senior high school and college level while almost equal distribution was seen among the junior high school level. Majority were fully vaccinated to COVID-19 with 1 booster dose. The presumed route of transmission was mostly from close contacts in school. Most common symptoms were cough, colds and fever. Most infections were mild. Among those vaccinated, the timing of infection from last vaccination showed varied results (9 to less than 12 months in junior high; 3 to less than 6 months and 6 to less than 9 months in senior high and college). The timing of recovery did not show significant difference (p-value=0.637) among different education levels. Based on the regression analysis, there is no variable which can be significantly associated with transmission of infection among close contacts either at school or at home. The highest number of cases and clustering were observed among medical and health-allied colleges and programs.

**Figure:**
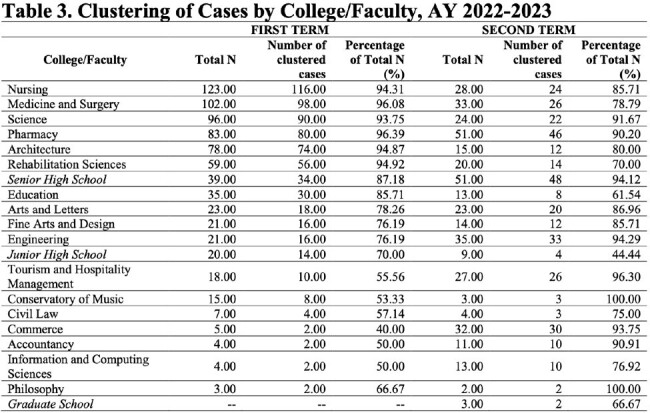

**Conclusion:**

Based on the study results, clustering can occur in school settings in the Philippines. Despite the low cases in the community, transmission is still present among university students. Therefore, infection prevention and control strategies must still be strictly observed until the virus is completely eradicated in order to prevent sporadic outbreaks.

**Figure:**
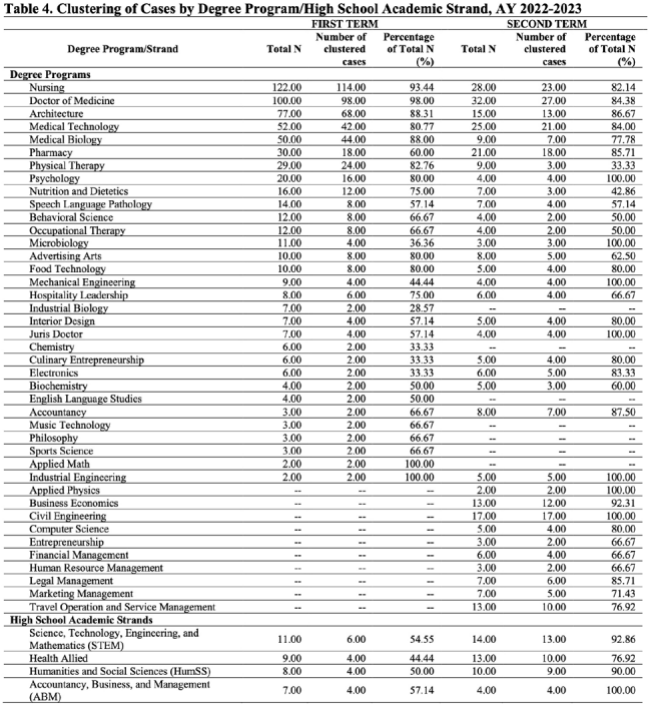

**Disclosures:**

All Authors: No reported disclosures

